# The first complete mitochondrial genome sequences of an ancient orphan evolutionary lineage of Eumolpinae leaf beetles endemic to the South Pacific

**DOI:** 10.1186/s12864-025-12170-z

**Published:** 2025-10-31

**Authors:** Anabela Cardoso, Jesús Gómez-Zurita

**Affiliations:** Botanical Institute of Barcelona (CSIC-CMCNB), Passeig del Migdia s/n, Barcelona, 08038 Spain

**Keywords:** Chrysomelidae, Codon usage, Database, Mitogenomes, Mitophylogenomics, Nucleotide composition bias

## Abstract

**Background:**

There are very limited mitogenome data representing most animal groups, particularly among the insects, which are otherwise extremely diverse and fulfill important ecological, sanitary, forest and agroeconomic roles. Increasing taxonomic diversity with new additions to the pool of mitogenomes for unrepresented evolutionary lineages is an opportunity to increase the phylogenetic power of mitogenome data, as well as refining our understanding of mitogenome diversity. Here, we characterize the complete mitogenomes of three species in two subgenera of *Taophila* leaf beetles, members of the hyperdiverse Eumolpinae, currently very poorly represented by mitogenomes, and the first representing an enigmatic independent evolutionary branch of the Eumolpini tribe, originated in the Cretaceous-Paleogene transition and endemic to the South Pacific.

**Results:**

These mitogenomes, assembled from genomic Illumina libraries, are relatively small (15,484–15,597 bp), retain the ancestral gene order of insect mitogenomes, except for a switch in the positions of *trnA* and *trnR*, a synapomorphic trait of the leaf beetle clade including Eumolpinae, Cryptocephalinae and Lamprosomatinae. Nucleotide composition, codon usage, relative synonymous codon usage and initiation and termination codons of protein-coding genes of these mitogenomes are all typical of insect mitogenomes, where nucleotide composition bias may be the result of mutation pressure, rather than natural selection. A mitogenome phylogeny of Eumolpinae, Cryptocephalinae and Lamprosomatinae revealed a strongly supported topology concordant with previous molecular systematic studies of these groups, including the identification of polytomies consistent with rapid early diversification of the Cryptocephalinae tribes and the separation of the main lineages within Eumolpini, one of them represented by *Taophila*.

**Conclusions:**

New mitogenomes from previously undersampled taxa contribute to an important, collective effort to populate mitogenome databases with an increasingly dense representation of the Tree-of-Life. It is crucial that these efforts are driven and supported by consolidated taxonomic expertise to guarantee the quality of results derived from these data. The availability of South Pacific Eumolpinae in the public mitogenome pool increases the likelihood of finding their missing link, if any, with other Eumolpini.

**Supplementary Information:**

The online version contains supplementary material available at 10.1186/s12864-025-12170-z.

## Introduction

The use of mitochondrial DNA (mtDNA) sequences for genetic and evolutionary studies in animals has a very long tradition [1]. But in the era of genomics, the ease of assembling complete or nearly complete mitogenomes as either byproducts or end-targets of genomic or metagenomic DNA sequencing using Next Generation Sequencing technologies has propelled the accumulation of mitogenome data for a large variety of organisms across the Metazoan tree of life (e.g., [2–4]). Currently, public sequence databases such as GenBank (28 November, 2024; https://www.ncbi.nlm.nih.gov/datasets/organelle/) host complete mitogenomes of 16,057 nominal species of animals, although they are not representative of Metazoan diversity, with nearly 49.0% of these data from chordates, and only 31.1% from insects, while these two animal classes represent approximately 3–6% and 75% of known species, respectively [5]. Moreover, there are also strong biases in species coverage within animal groups. For example, based on these same data, in the case of underrepresented insects, some 21.6% of available reference mitogenome data actually correspond to different species of Lepidoptera, while a mere 14.0% and 5.1% represent the inordinately diverse beetles and hymenopterans, with some 702 and 256 species with characterized mitogenomes (between 1.5–2.0‰ of described species), respectively. The precision of interpretations based on GenBank data mining may be affected by the vagaries of mining strategies but also annotation and taxonomic inaccuracy, but support to this bias comes from an independent, detailed review of insect mitochondrial genomics by S. L. Cameron [4], reaching similar estimates and conclusions based on data collected one year prior to ours. Data accumulates rapidly, but there are still huge gaps in our representation of the animal tree of life when it comes to mitogenomes. In this respect, mitophylogenomics, phylogenetic studies based on the full or most information available in mitogenomes, has proven a useful approach to increase the diversity of available mitogenomes, because of their interest in representing taxonomic diversity within and around the focal taxonomic group (e.g., [6–10]). At the same time, the utility of mitophylogenomics is compromised by uneven taxon coverage and, as these data become comparable in terms of taxonomic representation to standard phylogenetic markers for most groups, the more power and support will have the results based on these approaches. Hybrid approaches that combine complete or partial mitogenomes and nuclear genes have provided increased phylogenetic support in some cases (e.g., [11, 12]). Acknowledging these biases and limitations highlights the importance of progressively filling the taxonomic gaps also for this type of data.

**Fig. 1:**
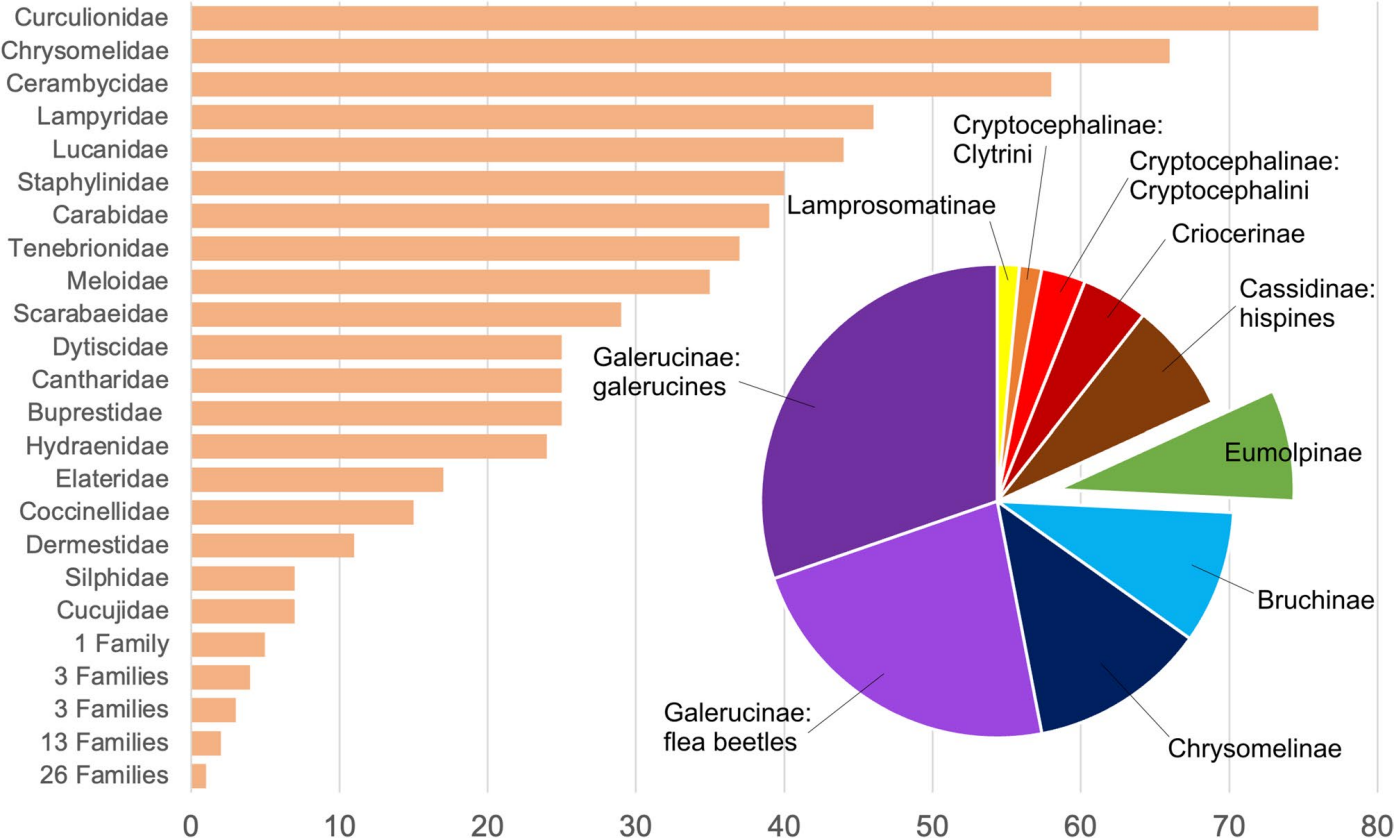
Reference mitogenomes of Coleoptera. Number of reference mitogenomes available for different families of Coleoptera in GenBank (November, 2024), and proportions of mitogenomes corresponding to different subfamilies (or suprageneric assemblages) of the Chrysomelidae. Eumolpinae, with five reference mitogenomes, and their closest relatives Cryptocephalinae and Lamprosomatinae, are poorly represented in public genome reference databases

When preparing this study, beetle mitogenomes tagged as reference genomes in GenBank—admittedly an underestimation, but taken as representative of existing data—are available for 65 families of Coleoptera, with 89% of these data belonging to 19 families only (Fig. 1). The bulk of these data were sequenced in dedicated mitophylogenomic studies of particular groups, such as fireflies (Lampyridae; [13]) or Onthophagini scarabs [14], or studies focusing on beetle species generally associated with stored products, agriculture or timber damage, including skin beetles (Dermestidae). The three largest phytophagous beetle families dominate available mitogenomes: Curculionidae, Chrysomelidae and Cerambycidae. With 65 reference mitogenomes (representing less than 1.6‰ of species in this family; [15]), leaf beetles are the second most sequenced beetle group, with many species studied due to their pest status, and a significant proportion have been characterized in the framework of mitophylogenomic studies (e.g., [16, 17]). Nie et al. [16]. focused on the largest subfamily of leaf beetles, the Galerucinae, representing more than half of available chrysomelid mitogenomes (Fig. 1), while other subfamilies have a low representation. Galerucinae and their sister group Chrysomelinae [18] include serious crop pests and this may explain, in part, why these subfamilies are the most common target of mitogenomic sequencing. For example, out of 52 leaf beetle species represented in the reference genome database in GenBank (3 December, 2024; https://www.ncbi.nlm.nih.gov/datasets/genome/), up to 44 were from species in these two subfamilies.

Eumolpinae stand out among the genomically less studied groups of leaf beetles, possibly because of their enormous diversity, with an estimated 7,000 described species [15], their mostly tropical distribution, and a general lack of taxonomic resources for the group. To date, there are complete mitochondrial genome sequences of Eumolpinae for five Asian species considered pests (Fig. 1), including *Aoria nigripes* (Baly, 1860) [19], *Basilepta fulvipes* (Motschulsky, 1860) [20] and *B. melanopus* Lefèvre, 1893 [21], *Colasposoma dauricum auripenne* (Motschulsky, 1860) [22], and *Trichochrysea japana* (Motschulsky, 1858) (Huang & Wu, unpublished data). Public nucleotide sequence databases, apart from a few unpublished sequences, also host nearly complete, partially annotated sequences for *Chalcophana sp.* [17], *Chrysochares punctatus* (Gebler, 1845) [23], *Chrysochus chinensis* Baly, 1859 [17], *Chrysodinopsis sp.* [24], *Platycorynus sp.* [17], and *Pseudocolaspis sp.* [25]. Several molecular phylogenetic studies have found a close association between Eumolpinae and the subfamilies Lamprosomatinae and Cryptocephalinae [17, 18, 26, 27], which are also poorly represented for genomic and mitogenomic resources (Fig. 1). A clade of these three subfamilies is also supported by a derived mitogenome arrangement [17], a synapomorphy consisting of the *trnR* and *trnA* genes having swapped positions relative to the arrangement found in the ancestral insect and retained by all other Chrysomelidae subfamilies sequenced to date.

The genus *Taophila* Heller, 1916 is an endemic genus of Eumolpinae from New Caledonia. This genus belongs to the tribe Eumolpini and is member of an enigmatic orphan lineage of this tribe endemic to and highly diversified in the South Pacific, from Vanuatu and New Caledonia to the South Island of New Zealand, also present in Fiji and Kermadec islands. The evolutionary enigma behind this South Pacific clade is the lack of clear shared ancestry with other groups, diverging from the rest of Eumolpini some 43.0–75.2 Ma ago [28]. One possible explanation for this orphan lineage is their origin in an evolutionary branch established in Antarctica but gone extinct at the end of the Eocene; however, sampling artifacts were not to be discarded [28]. *Taophila* has 21 species, one in the subgenus *Jolivetiana* Gómez-Zurita & Cardoso, 2014, and the rest in the nominotypical subgenus [29]. The genus has some rather interesting biological attributes for evolutionary and ecological studies, including striking sexual dimorphism, with females displaying the most distinctive morphological traits among species [29]. *Taophila* also includes one of the few documented cases of trophic associations with ferns in the leaf beetles [30, 31]. As mentioned, previous molecular systematics attempts at finding the evolutionary ties of South Pacific Eumolpini with other relatives worldwide were unsuccessful [28]. Considering that taxonomic density for mitophylogenomic studies will increase more rapidly than nuclear or genomic information [4], the availability of high-quality mitogenomic data for this lineage shall increase the chances of eventually finding their closest relatives, the missing link, or support the hypothesis of extinct relatives, possibly in Antarctica. To this end, in this work, we contribute complete, fully-annotated mitochondrial genomes for three species in both subgenera of *Taophila*. These data add one evolutionary branch previously missing from the mitophylogenomic tree of the Eumolpinae. Nucleotide composition and codon usage features of these new mitogenomes are described and the new mitogenomic data used to infer the phylogenetic history of the Eumolpinae, Cryptocephalinae and Lamprosomatinae.

## Materials and methods

### Samples and sequencing

Non-destructive genomic DNA extractions obtained with the DNeasy Blood & Tissue kit (Qiagen Iberia, Madrid) from a previous study [31], were available for three species of *Taophila*, including the type species, *T. subsericea* Heller, 1916 (specimen JGZC-NC103), *T. bituberculata* Platania & Gómez-Zurita, 2022 (specimen JGZC-NC102) and *T. mantillerii* Jolivet, Verma & Mille, 2007 (specimen JGZC-NC091), the type of the monotypic subgenus *Jolivetiana* Gómez-Zurita & Cardoso. Library preparation and sequencing were externalized to the Sequencing Unit at CNAG (National Center for Genomic Analyses, Barcelona, Spain). Extracted DNA was quantified using Qubit (Thermo Fisher Scientific Inc., Waltham MA) and Femto Pulse (Agilent Technologies Spain, Madrid) technologies, yielding total DNA amounts between 0.18 and 0.52 µg. Genomic DNA was sheared on a LE220-Plus Focused ultrasonicator (Covaris Ltd., Brighton, UK) and short-insert paired-end libraries were obtained with the PCR-free protocol of KAPA HyperPrep Kit (Roche Sequencing Solutions, Inc., Pleasanton CA), including end-repair, adenylation and ligation to Illumina-compatible adaptors with unique dual indexes and unique molecular identifiers (Integrated DNA Technologies, Leuven, Belgium). Sequencing was performed in paired-end mode (2 × 151 bp) on two independent flow cells on a NovaSeq 6000 Sequencing System (Illumina, San Diego CA), and raw data processing, including image analysis and FASTQ generation, were done with the manufacturer’s Real-Time Analysis software, RTA version 3.4.4. Data obtained from each flow cell were analyzed independently to assess the performance of the assembly and annotation procedure. Voucher specimens and DNA aliquots are kept in the authors collections (IBB, CSIC, Spain) and raw sequence data are available under the European Commission Open Data Repository, Zenodo (10.5281/zenodo.16900841).

### Mitogenome assembly and annotation

Data quality control and filtering, assembly and annotation were performed with MitoZ version 3 [32]. MitoZ is a Python pipeline that completes raw paired-end reads filtering and cleaning with a custom Perl script; read assembly with MEGAHIT [33]; filtering of false mitochondrial scaffolds and NUMTs based on HMMER, a profile Hidden Markov Model approach [34] and Tiara classification of sequences [35]; and annotation using tBLASTn and Genewise [36] for protein-coding genes (PCG), MiTFi [37] for tRNAs, and Infernal [38] for rRNAs. MitoZ assembly and annotation used software defaults, except that a maximum of 4 or 5 Gb for raw data (equivalent to 26.49 M or 33.11 M reads) were used in assemblies, “Arthropoda” was specified as taxonomic source to guide the selection of correct mitochondrial genome scaffolds and “Coleoptera” for annotation steps. A multi-Kmer approach for genome assembly was applied with K = 79, 99 and 119. Complete, circularized mitogenomes inferred by MitoZ have random initiation points, and sequences were reoriented to a standard gene order manually in Geneious Prime version 2024.0.7 (Dotmatics, Boston MA). The complete annotated mitogenomes of *Taophila* were deposited in GenBank under accession numbers PV240303 (*T. bituberculata*), PV240304 (*T. mantillerii*) and PV240305 (*T. subsericea*).

### Analysis of protein-coding gene features

Structural features of annotated mitogenomes, including gene length, nucleotide composition, and start/stop codons, were extracted in Geneious Prime. Alternative start and stop codons and incomplete stop codons lack empirical verification, and were deduced computationally and based on comparisons with published mitogenome annotations of close relatives. Codon usage bias was estimated via relative synonymous codon usage (RSCU) metrics, from a concatenation of the protein-coding genes using CAIcal [39], and the effective number of codons metric (Nc; [40]) with CodonW version 1.4.4 (written by John Peden, and implemented in the Galaxy server; [41]). Nucleotide composition bias was estimated via correlation of G + C content at combined first and second codon positions (GC12) versus third codon positions (GC3); this correlation can distinguish the role of mutation pressure versus natural selection in nucleotide composition [42]. The fit of these two variables to normal distributions was assessed based on the Shapiro-Wilk normality test and homoscedasticity on the Breusch-Pagan test. The significance of correlation was estimated using the Pearson correlation test. All tests were computed with the ggplot2, ggpubr and car packages in R version 4.3.1 [43].

### Mitophylogenomics

Complete or nearly complete annotated mitogenomes of Cryptocephalinae, Lamprosomatinae and Eumolpinae were obtained from GenBank, together with the mitogenomes of representatives of two subfamilies traditionally associated with Eumolpinae, and considered sister to the clade composed of the three subfamilies of interest [18, 27, 44], selected here as outgroups: *Spilopyra sumptuosa* Baly, 1860 (Spilopyrinae; [17]) and *Syneta adamsi* Baly, 1877 (Synetinae; [17]). Sequences availability is listed in Table 1. The 13 protein-coding and two rRNA genes of these leaf beetle mitogenomes were aligned independently using MAFFT 7 algorithms, L-INS-i for protein-coding genes and Q-INS-i for rRNA genes [47]. Ragged ends in some alignments (*nad1*, *nad3* and rRNA genes) were removed manually prior to the concatenation of individual alignments. The concatenated matrix was analyzed under maximum likelihood in RAxML NG [48] and Bayesian inference in MrBayes version 3.2.7 [49] with unlinked independent GTR + I + G substitution models for rRNA genes, third codon positions of protein-coding genes, and combined first and second codon positions of these genes, for a total of 28 independent data partitions. Maximum likelihood phylogenetic inference consisted of 20 individual runs starting from random topologies, with branch support (BS) assessed through bootstrap search with 500 pseudoreplicates. Bayesian inference consisted of two independent runs of one cold and three heated chains (default temperature = 0.2) for 15 M generations each, sampling parameters and trees every 1,000 generations (average standard deviation of split frequencies between runs dropped below 1% after 700 K generations). Tree topologies and node posterior probabilities (PP) were summarized excluding the initial 25% of saved trees. The optimal maximum likelihood tree and the maximum clade credibility Bayesian tree were rooted secondarily to the outgroups, Spilopyrinae and Synetinae.

**Table 1:** Taxon sampling for the mitophylogenomic analysis of the Eumolpinae clade and closest relatives

Subfamily	Species	Accession	Reference
Cryptocephalinae			
Fulcidacini	*Chlamisus* sp.	MK049868	[17]
Clytrini	*Chilotomina oberthuri*	KX943432	[45]
	*Clytra espanoli*	KX943426	[45]
	*Clytra quadripunctata*	KX943461	[45]
	*Labidostomis ghilianii*	KX943477	[45]
	*Labidostomis lucida*	KX943488	[45]
	*Labidostomis urticarum*	MK049877	[17]
	*Lachnaia cylindrica*	KX943427	[45]
	*Lachnaia gallaeca*	KX943443	[45]
	*Lachnaia hirta*	KX943433	[45]
	*Peploptera acromialis*	HQ232809	[46]
	*Physosmaragdina* sp.	MK049873	[17]
	*Smaragdina concolor*	KX943498	[45]
	*Smaragdina reyi*	KX943492	[45]
Cryptocephalini	*Adiscus speciosus*	NC_066821	Unpublished
	*Cryptocephalus androgyne*	KX943411	[45]
	*Cryptocephalus aureolus*	KX943487	[45]
	*Cryptocephalus dimidiatipennis*	NC_066822	Unpublished
	*Cryptocephalus flavolimbatus*	MK049867	[17]
	*Stylosomus ilicicola*	KX943463	[45]
	*Stylosomus rugithorax*	KX943428	[45]
Pachybrachini	*Pachybrachis suffrianii*	KX943489	[45]
Eumolpinae			
Bromiini	*Aoria nigripes*	NC_065028	[19]
	*Bromius obscurus*	KX087249	Unpublished
	*Pseudocolaspis* sp.	KY039144	[24]
	*Pseudocolaspis* sp.	KY039145	[24]
	*Pseudocolaspis* sp.	JX412756	[25]
Euryopini	*Colasposoma dauricum*	KY039104	[24]
	*Colasposoma* sp.	KY039108	[24]
Eumolpini	*Chalcophana* sp.	MK049868	[17]
	*Chrysochares punctatus*	MN745103	[23]
	*Chrysochus chinensis*	MK049871	[17]
	*Chrysodinopsis* sp.	KY039111	[24]
	*Platycorynus* sp.	MK049872	[17]
	*Taophila bituberculata*	PV240303	This study
	*Taophila mantillerii*	PV240304	This study
	*Taophila subsericea*	PV240305	This study
Typophorini	*Basilepta fulvipes*	MT627597	[20]
	*Basilepta melanopus*	OP115728	[21]
	*Trichochrysea japana*	OR387477	Unpublished
Lamprosomatinae	*Oomorphoides metallicus*	MK085754	[17]
Spilopyrinae	*Spilopyra sumptuosa*	MK049875	[17]
Synetinae	*Syneta adamsi*	MK049876	[17]

## Results

### Characteristics of Taophila mitogenomes

**Fig. 2:**
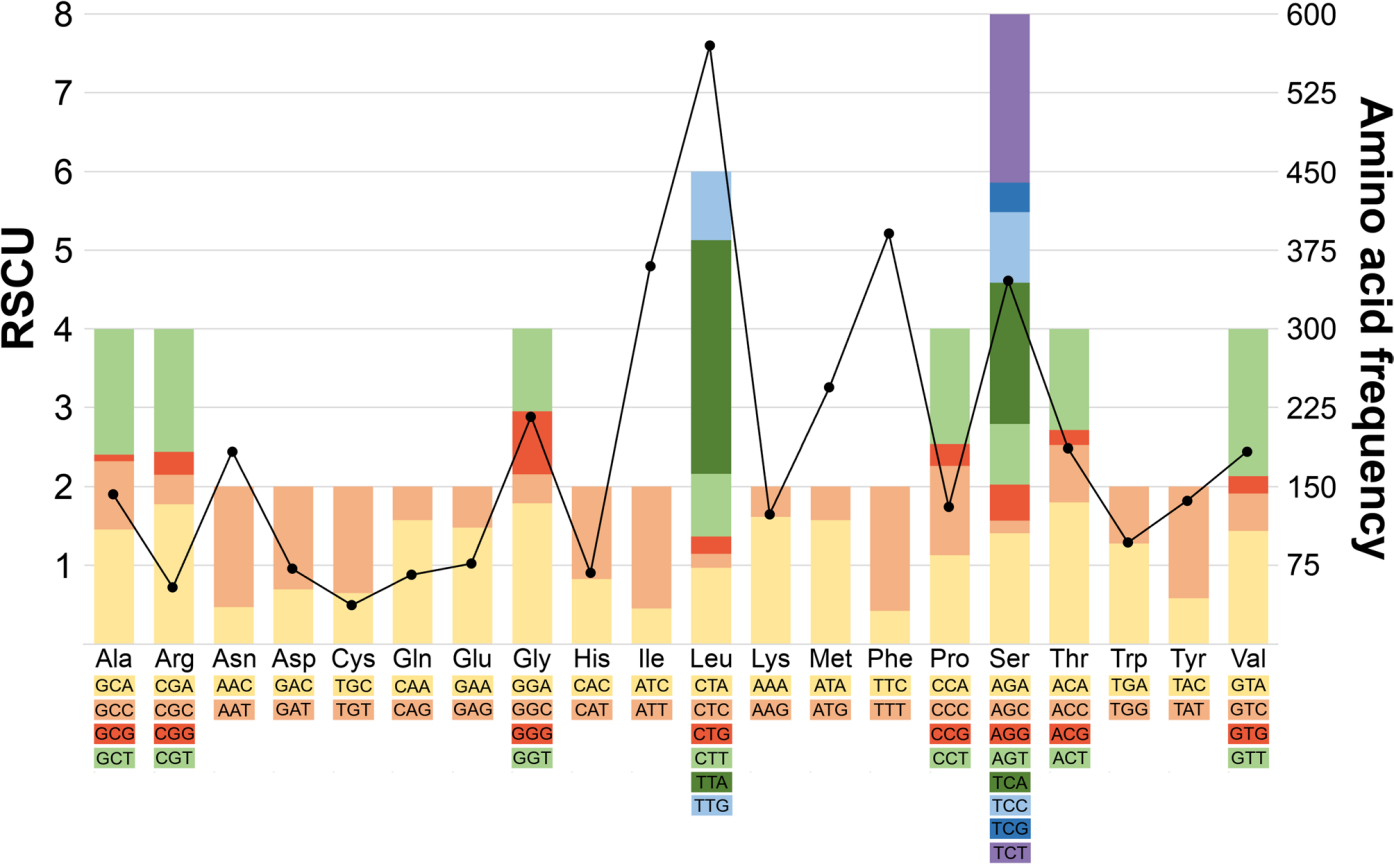
Complete mitogenome of *Taophila subsericea* Heller

The MitoZ pipeline used approximately 19–61% of the raw read content of the original libraries, which provided nonetheless abundant high-quality sequence data for mitogenome assembly in every case, both in terms of contigs representing this genome compartment and the read depth per individual position of the assembled mitogenomes (Table 2). Duplicate mitogenome assemblies of different Illumina runs at the same individual’s DNA produced identical assembled sequences in all cases, except for differences in circularization in the control region, showing a duplication of a tract of 36 bp in one *T. mantillerii* assembly, of 50 bp in one *T. subsericea* assembly, and a tandem duplication of this same segment, adding 100 bp in one assembly of *T. bituberculata* (Table 2). By removing these artefactual duplications, the genomes were 15,597 bp in length in *T. mantillerii*, 15,498 bp in *T. subsericea*, and 15,484 bp in *T. bituberculata*. Gene order and orientation of all three *Taophila* mitogenomes were identical to those found in the published mitogenomes of other Eumolpinae, Cryptocephalinae and Lamprosomatinae (Fig. 2).

**Table 2:** Illumina library and *Taophila* mitogenome assembly characteristics, including data on number and quality of reads, assembled contigs and inferred genome source (based on Tiara), and sequencing depth

	T. bituberculata		T. mantillerii		T. subsericea	
	Library 1	Library 2	Library 1	Library 2	Library 1	Library 2
Library reads (M)	43.33	138.34	48.31	139.81	55.87	125.02
Total reads used (M)^a^	26.49	33.11	26.49	26.49	26.49	33.11
Reads filtered (M)	26.18	32.78	25.94	25.92	26.20	32.77
Q30	93.64	93.44	93.49	93.02	94.25	93.76
Insert size peak (bp)	267	266	151	151	268	262
Megahit Contigs	21,603	24,510	17,780	22,661	25,412	32,872
Eukarya	18,052	20,415	14,610	18,589	21,048	27,201
Organelle	801	898	661	901	1013	1339
Mitochondrion	218	237	195	265	263	367
Minimum depth^b^	189	231	3	35	587	807
Maximum depth^b^	3207	5721	3796	3535	5516	6173
Average depth^b^	666.5 ± 255.23	845.8 ± 395.73	674.5 ± 170.53	680.7 ± 156.51	890.6 ± 208.54	1121.6 ± 221.92
Length	15,584	15,484	15,633	15,597	15,498	15,548

^a^ In two cases, MitoZ failed to circularize the mitogenomes, and the problem was solved by increasing the amount of initial data, from 4 to 5 Gb, the maximum allowed by the pipeline

^b^ The extremes were always found in the control region, and depth average values were representative of the whole coding region

### Characteristics of protein-coding genes

**Fig. 3:**
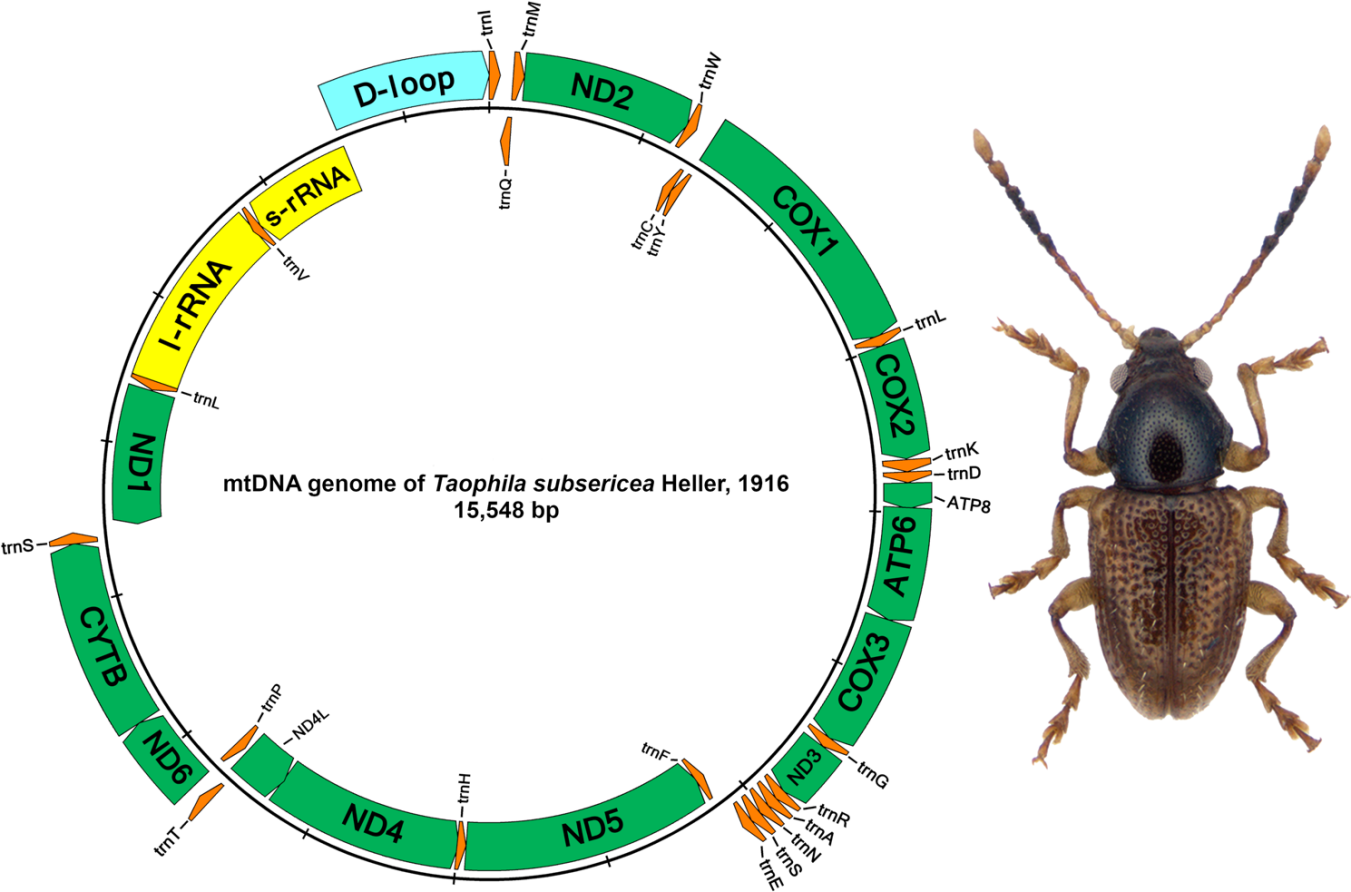
Codon usage in the mitogenome of *Taophila*. Stacked column chart representing relative synonymous codon usage of the mitogenome of *Taophila subsericea* Heller, and line graph showing the absolute frequency of each amino acid in the set of protein-coding genes in this mitogenome

The absolute proportions of amino acids coded for in protein-coding genes of the mitogenome of *Taophila subsericea*, as well as the RSCU for each amino acid are shown in Fig. 3. The other *Taophila* mitogenomes show the same pattern with small variations (Fig. S1). Just five of 20 amino acids represented more than half (51.9%) of all the coded amino acids in the mitogenome: the polar uncharged Serine, and the hydrophobic Leucine, Phenylalanine, Isoleucine and Methionine. The five least used amino acids (8.0%) were the electrically charged Aspartic Acid, Arginine and Histidine, and the polar Glutamine and special Cysteine. The most utilized start codons in *Taophila* were ATN (except ATA), but also TTG, used by *nad1*-*nad3*. The most common stop codon was TAA, although TAG or truncated stop codons (T– and TA-) were identified as well (Table 3). Synonymous codon usage was always biased towards codons ending in an A or T base (Fig. 3). The G + C contents of third (W = 0.96939, p-value = 0.360) and combined first and second (W = 0.94678, p-value = 0.064) codon positions fitted normal distributions (Fig. S2), and the variances of both variables were similar (Chi-Squared = 0.21597, d.f. = 1, p-value = 0.642). The correlation between GC12 and GC3 (Fig. S2) was significant (correlation coefficient = 0.4358, t = 2.9451, d.f. = 37, p-value = 0.0056).

**Table 3:** General characteristics of protein coding genes of the mitogenomes of three species of *Taophila* leaf beetles

Gene	Length	AA	%GC^a^	GC3^a^	Start s. str.	Start Jolivetiana	Stop s. str.	Stop Jolivetiana
*atp6*	675	224	27.4/28.6/31.1	20.1/20.1/23.7	ATG (M)		TAA	
*atp8*	156	51	18.6/19.2/23.1	11.8/17.6/15.7	ATT (I/M)		TAA	
*cox1*	1545	514	35.0/37.5/34.5	22.8/29.4/20.4	ATT (I/M)		TAA	
*cox2* ^b^	684	227	29.7/31.6/32.0	15.0/20.7/19.2	ATT (I/M)	ATC (I/M)	TAA	T--
*cox3*	785	261	30.3/34.0/33.2	14.2/27.6/25.7	ATG (M)		TA-	
*cytb*	1140	379	31.1/35.7/32.2	21.1/30.6/20.8	ATG (M)		TAA	
*nad1*	951	316	25.2/28.4/26.0	13.6/22.5/17.7	TTG (L/M)		TAG	
*nad2*	1011	336	25.9/26.8/27.4	20.2/21.4/22.3	TTG (L/M)		TAA	
*nad3*	354	117	26.0/29.1/27.7	21.4/29.1/23.9	TTG (L/M)	ATC (I/M)	TAA	
*nad4*	1327	442	22.5/26.5/25.1	13.3/22.6/20.8	ATG (M)		T--	TAG
*nad4l*	288	95	22.6/22.6/24.0	18.9/18.9/17.9	ATG (M)		TAA	
*nad5*	1714	571	22.6/26.0/23.5	11.9/20.0/13.8	ATT (I/M)		T--	
*nad6*	489	162	21.1/24.5/21.9	16.7/22.8/17.3	ATT (I/M)	ATC (I/M)	TAA	

^a^ Total and third codon position G + C content is given for *T. bituberculata/T. subsericea/T. mantillerii*

^b^ In *T. mantillerii*, the annotation extended the *cox2* gene 4 nt and 2 extra aa’s

The effective number of codons (Nc) per gene showed a strong to moderate bias, with values ranging between 30.49 and 46.51 in *T. bituberculata*, 32.79 and 42.19 in *T. mantillerii*, and 29.35 and 47.60 in *T. subsericea* (Table 4). In all three mitogenomes, NADH dehydrogenase subunit genes typically showed more biased values compared to the other genes.

**Table 4:** Effective number of codons in protein-coding genes of three mitogenomes of *Taophila*

Gene	T. bituberculata	T. mantillerii	T. subsericea
*atp6*	36.41	41.36	39.38
*atp8*	46.51	-	29.35
*cox1*	41.93	38.83	43.41
*cox2*	38.16	40.45	42.98
*cox3*	37.37	39.70	47.60
*cytb*	37.13	38.41	44.36
*nad1*	34.97	34.05	38.40
*nad2*	37.83	42.19	39.61
*nad3*	32.04	38.19	37.37
*nad4*	30.49	35.05	38.92
*nad4l*	32.37	38.02	34.91
*nad5*	30.95	36.26	34.67
*nad6*	35.15	32.79	39.90

## Mitophylogeny of the Eumolpinae clade

**Fig. 4:**
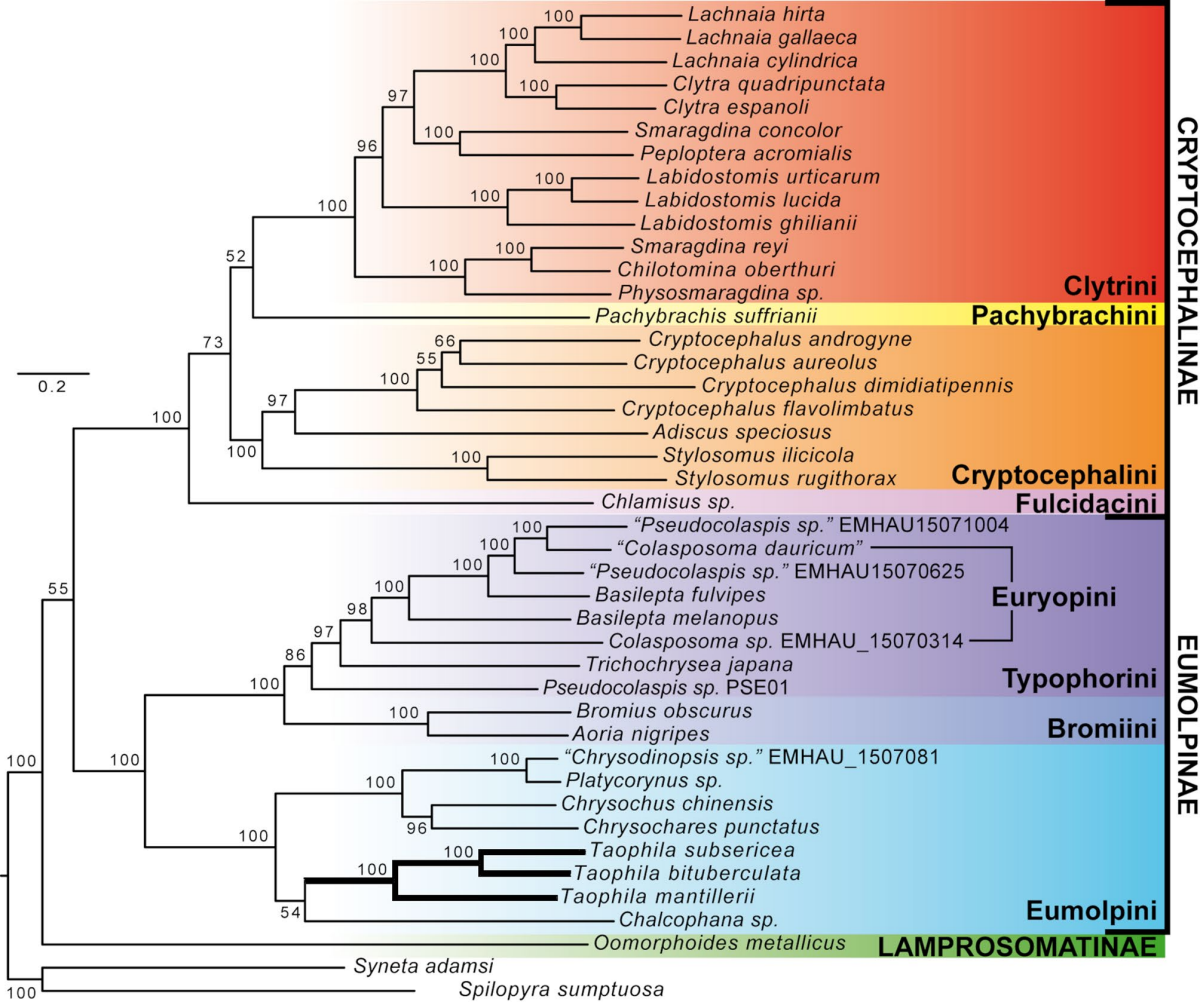
Mitophylogenomics of the Eumolpinae clade. Optimal maximum likelihood tree (L = −282,375.230644) based on protein-coding genes and rRNA data from the mitogenomes of Cryptocephalinae, Eumolpinae and Lamprosomatinae. Branch support is shown as bootstrap percentages, the lineage of new mitogenomes shown with thickened branches, and clades consistent with the subfamily and tribe systematics of leaf beetles are highlighted

The mitophylogeny of Eumolpinae and closest relatives was consistent with previous hypotheses of relationships in this group, where the three subfamilies formed a basal polytomy (Figs. 4 and S3). The Cryptocephalinae were well supported (BS = 100%, PP = 1.00) with moderate support (BS = 73%) in the maximum likelihood tree for the Fulcidacini as sister of the remaining tribes, which formed a polytomy of monophyletic Cryptocephalini (BS = 100%), Pachybrachini (monotypic) and Clytrini (BS = 100%). In the Bayesian tree, these relationships were fully resolved as (Fulcidacini, (Pachybrachini, (Cryptocephalini, Clytrini))) (Fig. S3). All genera within Cryptocephalinae with more than one species sampled were monophyletic, with the exception of *Smaragdina*, also consistent with previous studies [18, 27]. Finally, the Eumolpinae clade was well supported (BS = 100%, PP = 1.00) with two strongly supported groups (BS = 100%, PP = 1.00), consistent with a clade of Bromiini, Typophorini and Euryopini, and one of Eumolpini, a topology consistent with previous phylogenies of this subfamily [18, 28, 44]. The genera *Basilepta*,* Colasposoma* and *Pseudocolaspis* in the first clade were not monophyletic. The new *Taophila* mitogenomes formed a monophyletic group (BS = 100%, PP = 1.00) within the Eumolpini clade, one of three well supported clades within a polytomy of Eumolpini lineages, in both the maximum likelihood and Bayesian trees.

## Discussion

Average GenBank reference mitogenome size in insects (accessed 28 November, 2024; https://www.ncbi.nlm.nih.gov/datasets/organelle/) is 15,801.0 bp (SD = 856.63 bp), whereby the smallest mitogenome characterized so far, lacking control region but including the typical set of 37 genes, belongs to a bird body louse, *Ciconiphilus decimfasciatus* (14,102 bp) [50], and the largest, owing to a huge control region and without genome duplications, to the netspinning caddisfly, *Potamyia horvati* (27,450 bp) [51]. There are smaller and larger mitogenome versions in insects, but they represent either fragmented genomes, as found in the slender duck louse, *Anaticola crassicornis* (8,118 bp) [52], or whole genome duplications, as described for the Australian stingless bee, *Tetragonula carbonaria* (30,666 bp) [53]. Apart from these profound rearrangements, insect mitogenome size is rather conserved and length variation can be mostly attributed to the control region. As a note of caution, assemblies obtained with nanopore long-read technology with enough sequence depth compared with Illumina short-read mitogenome assemblies, have shown that the latter can potentially collapse repetitive elements, thus resulting in underestimated mitogenome sequenced lengths (e.g., [54]). With an average length of 16,442 bp (SD = 986.9 bp), beetle mitogenomes fall within the insect size range, but with less size variation: from the 14,476 bp of the cigarette beetle, *Lasioderma serricorne* [55], to the 22,714 bp in a European stag beetle, *Lucanus cervus* (Y. Ying, unpublished data). The mitogenomes of *Taophila* would be in the lowest 13.8% of this distribution, thus they can be classified as small insect mitogenomes, mainly owing to a relatively short origin of replication (857–1,021 bp). As stated above, most length variation of insect mitogenomes can be attributed to this non-coding region, and this is logically true for beetle mitogenomes as well, which can reach up to 5,000–6,000 bp in some species (e.g., [56, 57]). Also contributing to the small size of *Taophila* mitogenomes, it is worth mentioning the limited and reduced intergenic spacers (for a total of 47 bp in *T. bituberculata* and *T. subsericea* and 59 bp in *T. mantillerii*) and some gene overlap (0.614% in *T. bituberculata*, 0.705% in *T. mantillerii*, and 0.716% in *T. subsericea*, of total genome size).

The structure, gene order and orientation of these new mitogenomes is identical to previously published mitogenomes of other eumolpines and their closest relatives, the cryptocephalines and lamprosomatines [17, 18, 27]. Any differences detected were confirmed to be due to tRNA or rRNA annotation mistakes in published mitogenomes, and included: (1) swapped order of *trnR* and *trnA*, and inclusion of D-loop in the *rrnS* in *Chrysochares punctatus* [23]; (2) inversion of *trnP* in *Colasposoma sp.* [24]; (3) inversion of *rrnS* in *Chrysodinopsis sp.* [24]; and (4) incorrect naming of the rRNA genes in *Chalcophana sp.*,* Chlamisus sp.*,* Chrysochus chinensis*,* Cryptocephalus flavolimbatus*,* Lachnaia urticarum*,* Physosmaragdina sp.*,* Platycorynus sp.*,* Spilopyra sumptuosa* and *Syneta adamsi* [17]. It is important to note that, even if gene identifications are correct for the cluster of tRNA genes between *nad3* and *nad5* genes in Nie et al. [17], both their Figs. 1 and 2 show *trnE* (Glutamate) inverted in the clade of Cryptocephalinae, Lamprosomatinae and Eumolpinae. Expectedly, *Taophila* mitogenomes include the novel order of the tRNA cluster between *nad3* and *nad5* that is synapomorphic for this clade [17]. Other than that, the characteristics of these new mitogenomes are the same as found in most Coleoptera and consistent with the inferred characteristics of the ancestral insect mitogenome [58]. From the point of view of diversity of tRNAs, *Taophila* mitogenomes show the same pattern as described for most Coleoptera and insects [6, 59, 60], presenting 22 tRNAs for the 20 amino acids, whereby the redundant *trnL* and *trnS* recognize the first and third most used codons in the *Taophila* mitogenomes. Most initiation and stop codons in *Taophila* are also typical of insect mitogenomes, including some incomplete T- or TA- termination codons completed to functional TAA stop codons by postranscriptional polyadenylation [61]. Interestingly, some of the *Taophila* NADH dehydrogenase subunit genes can start with the unusual TTG (Leu) initiation codon, first reported from insects in a springtail [62], and later found in other insect mitogenomes, but nonetheless present at very low frequences and only for a few genes [63]. Codon usage proportions in the mitogenomes of *Taophila* are very similar to those deduced from other leaf beetles, with minor differences. For example, Arginine seems underutilized compared to *Gastrolina* chrysomelines [64], or Valine more frequently used when compared to *Luperomorpha* flea beetles [65], but the general pattern of amino acid use is highly conserved. Finally, RSCU analysis reveals a bias towards codons ending in A or T, another common feature of insect mitogenomes (e.g [66]). This bias is also reflected in the effective number of codons per gene, indicative of a subset of codons used preferentially, i.e., with A or T at third codon positions. In combination with RSCU data, the significant correlation between GC-content at third versus first and second codon positions (p-value = 0.0056) suggests uniform base use in these mitogenomes. Codon usage bias can thus be interpreted as predominantly due to mutation pressure rather than selection [42, 67]. Neutrality plot analysis is a common way to assess the alternative roles of these two forces acting on codon usage bias, with empirical results from different groups of insects sometimes supporting mutational bias, as it occurs in *Taophila* (e.g., [68, 69]), and sometimes the alternative of selection (e.g., [70, 71]).

Mitophylogenomics of eumolpines and relatives reveals phylogenetic relationships generally consistent with the current systematics of these groups [17, 27], where most nodes (82.9%) receive strong bootstrap support (BS = 95–100%), and all but four nodes the highest posterior probabilities (PP = 0.98–1.00.98.00). Nodes which lack support (BS = 52–73%) from mitogenome data also failed to be resolved in studies with denser taxonomic sampling and data from several single-copy nuclear protein-coding genes, suggesting that they could reflect “hard” polytomies, i.e., rapid diversification of those clades rather than a polytomy due to conflict or lack of resolving data [72, 73]. These polytomies include the relationships among the tribes in Cryptocephalinae (although Bayesian inference also lent some support for example to a closer relationship between Clytrini and Cryptocephalini; [27]), and also among evolutionary lineages in Eumolpini, one of which is the South Pacific lineage represented by *Taophila* [28]. More problematic, in this case, is the apparent lack of monophyly for genera represented by more than one species in the Eumolpinae clade, or the placement of some species (such as *Chrysodinopsis sp.*). However, we suggest that these unexpected placements are not due to low resolving power of mitogenomes to solve a systematic problem, but due to the deficient taxonomy in most groups of Eumolpinae today [74]. Two intimately related problems possibly produce the seemingly scrambled taxonomy found in our mitochondrial phylogenetic hypothesis. First, the uncertain underlying taxonomy and lack of modern revisions of the huge genera *Basilepta*,* Colasposoma* and *Pseudocolaspis*, do not exclude that they may be indeed paraphyletic groups. Secondly, the lack of taxonomic expertise in Eumolpinae hampers sound taxonomic identifications in many cases. Both factors greatly reduce our confidence in the identifications attached to some sequences, and those characterized by Song et al. [24] are particularly problematic. Samples from this study generate all the conflicts observed at the generic level in the tree (despite these samples clearly having correct tribal identifications). Unfortunately, these sequences lack public metadata that could help in tracking their origin, and represent Afrotropical and New World taxa that cannot be reliably identified with the Chinese faunistic references used by the authors [24].

Mitogenomes continue to offer ample advantages for their implementation in evolutionary studies, including practical issues such as the relative ease in their sequencing with NGS methodologies and annotation via dedicated analytical pipelines [4, 45]. The deep knowledge about the structure, function and dynamics of this molecule, also helps exploiting mitogenomic information, as well as spotting interesting peculiarities worth further investigation, such as gene rearrangements [4]. Increasing the taxonomic quality and density of mitogenome data, especially targeting evolutionary lineages not represented in the available mitogenomes, will benefit the formulation of phylogenetic hypotheses useful for systematic and other evolutionary studies. This trend and the addition of *Taophila* to the mitogenome pool will be crucial to eventually resolve the phylogenetic position of South Pacific Eumolpinae. This lineage, currently lacking known closest relatives, may prove highly relevant to incorporate Antarctica to biogeographical models of the Southern hemisphere, as suggested for other Chrysomelidae, such as Chilean Mylassini cryptocephalines [27]. Alternatively, the steady addition of mitogenomes from pooled or environmental samples (e.g., [75, 76]), may help revealing, if by chance, an extant missing link of South Pacific Eumolpinae and support a more conventional biogeographic hypothesis. But in this respect, it is important that the publication of new mitogenomic resources is supported by robust identifications and include taxonomic details of analyzed samples, such as metadata to allow not only comparisons derived from these data, but also tracing the origin of samples for their reassessment (e.g., [77, 78]).

## Supplementary Information

Supplementary Material 1

## Data Availability

The specimens providing the DNA for this study are part of the Chrysomelidae research collection of the senior author (CSIC, Spain), with voucher numbers JGZC-NC103 (*T. subsericea*), JGZC-NC102 (*T. bituberculata*) and JGZC-NC091. Raw Illumina sequencing data used for this study are deposited in the Zenodo DOI: 10.5281/zenodo.16900841. As most data have not yet been analyzed by data producers, we request further data analysts to contact the authors before planning their use and always citing this article as the original source of these data. The assembled and annotated mitogenome data generated and analyzed for this study are available in the European Nucleotide Archive repository, under accession numbers PV240303–PV240304. The phylogenetic matrix assembled for the mitophylogenomic study of the Eumolpinae clade is available through the Zenodo DOI: 10.5281/zenodo.14987316.
