# Supplementary material for: The first complete mitochondrial genome sequences of an ancient orphan evolutionary lineage of Eumolpinae leaf beetles endemic to the South Pacific
Source: BMC Genomics. 2025 Oct 31;26:982. doi: 10.1186/s12864-025-12170-z (PMC12577182; doi:10.1186/s12864-025-12170-z)
Supplement: Supplementary file 1 — Supplementary Material 1 [file 12864_2025_12170_MOESM1_ESM.pdf]

Supplementary Information

The first complete mitochondrial genome sequences of an ancient evolutionary lineage of Eumolpinae leaf beetles endemic to the South Pacific

Anabela CARDOSO & Jesús GÓMEZ-ZURITA

Botanical Institute of Barcelona (CSIC-CMCNB), Passeig del Migdia s/n, 08038 Barcelona, Spain.

Figure S1. Stacked column charts representing relative synonymous codon usage of the mitogenome of *Taophila bituberculata* Platania & Gómez-Zurita (a) and *T. mantillerii* Jolivet, Verma & Mille (c), and line graphs showing the absolute frequency of each amino acid in the set of protein-coding genes in these same two mitogenomes (b: *T. bituberculata*; d: *T. mantillerii*).

Figure S2. Quantile-quantile plots of the variables relative to GC content in first and second (a) or third (b) codon positions consistent with these variables fitting normal distributions, and correlation between these two variables (c) in the mitogenome of *Taophila subsericea*.

Figure S3. Maximum clade credibility Bayesian tree of the complete set of protein-coding and rRNA genes from mitogenomes of Cryptocephalinae, Eumolpinae and Lamprosomatinae leaf beetles. Average arithmetic and harmonic means of likelihood values for two independent runs of the analysis were -277,968.02 and -278,072.08, respectively. Clade posterior probabilities were 1.00 for all nodes, except where indicated otherwise.

Figure S1

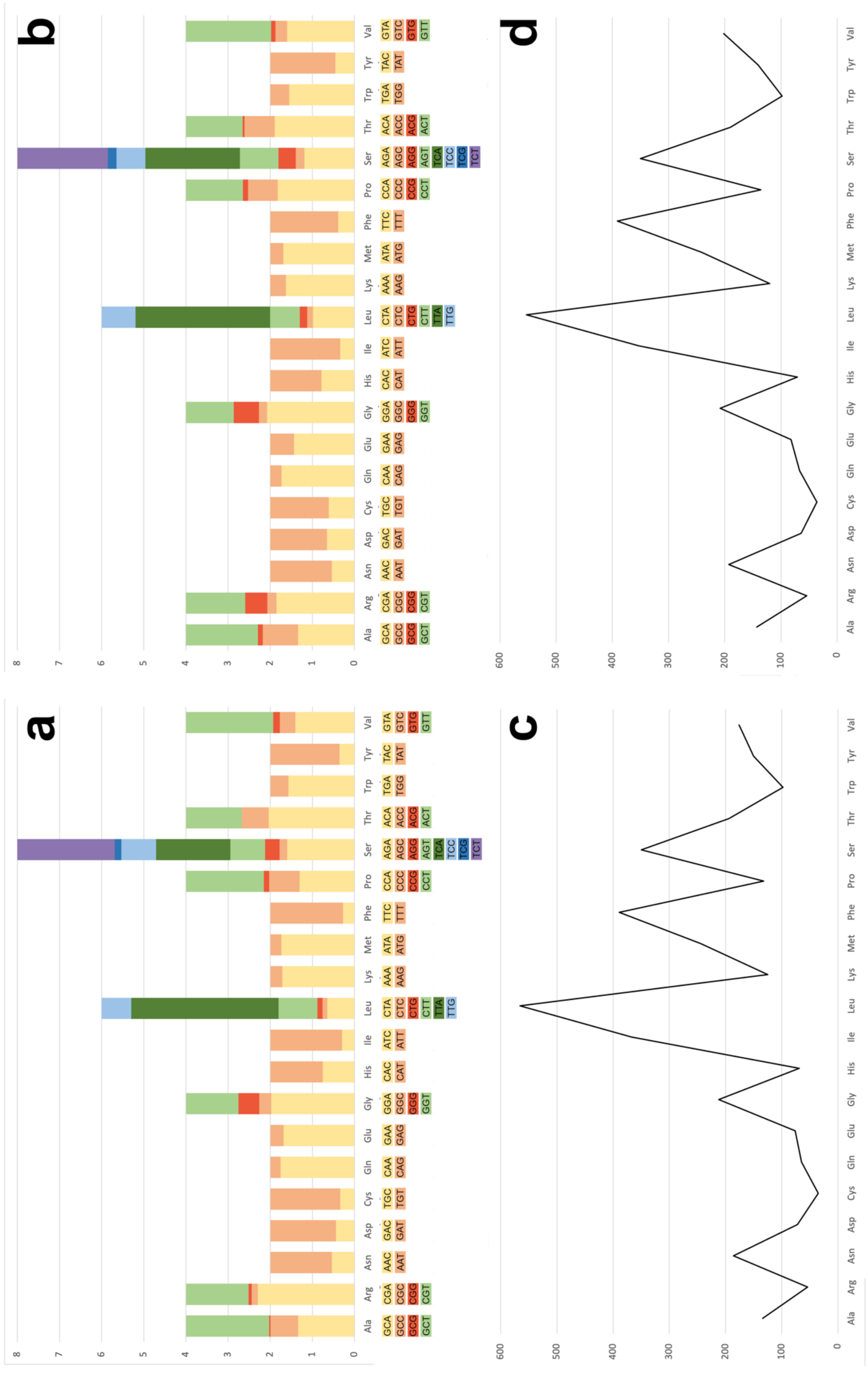

a
b
c
d
Ala
Arg
Asn
Asp
Cys
Gln
Glu
Gly
His
Ile
Leu
Lys
Met
Phe
Pro
Ser
Thr
Trp
Tyr
Val

Figure S2

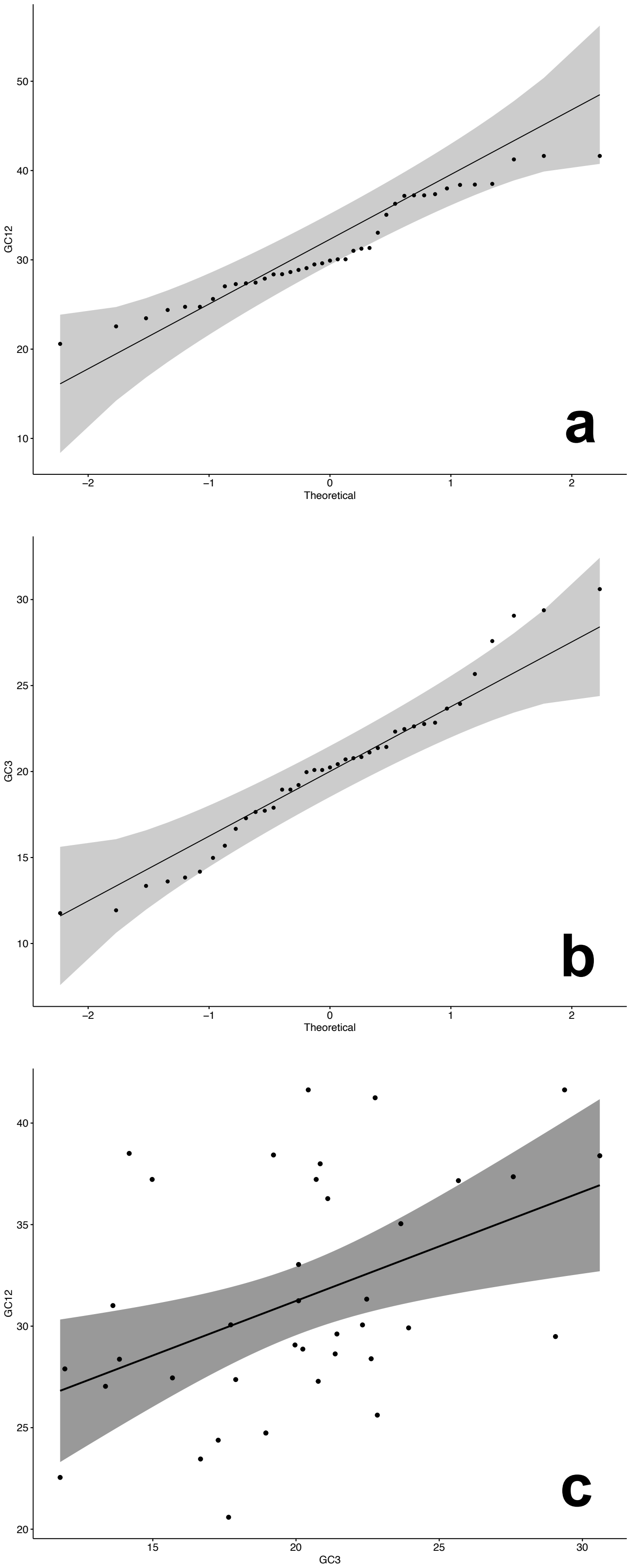

Figure S3

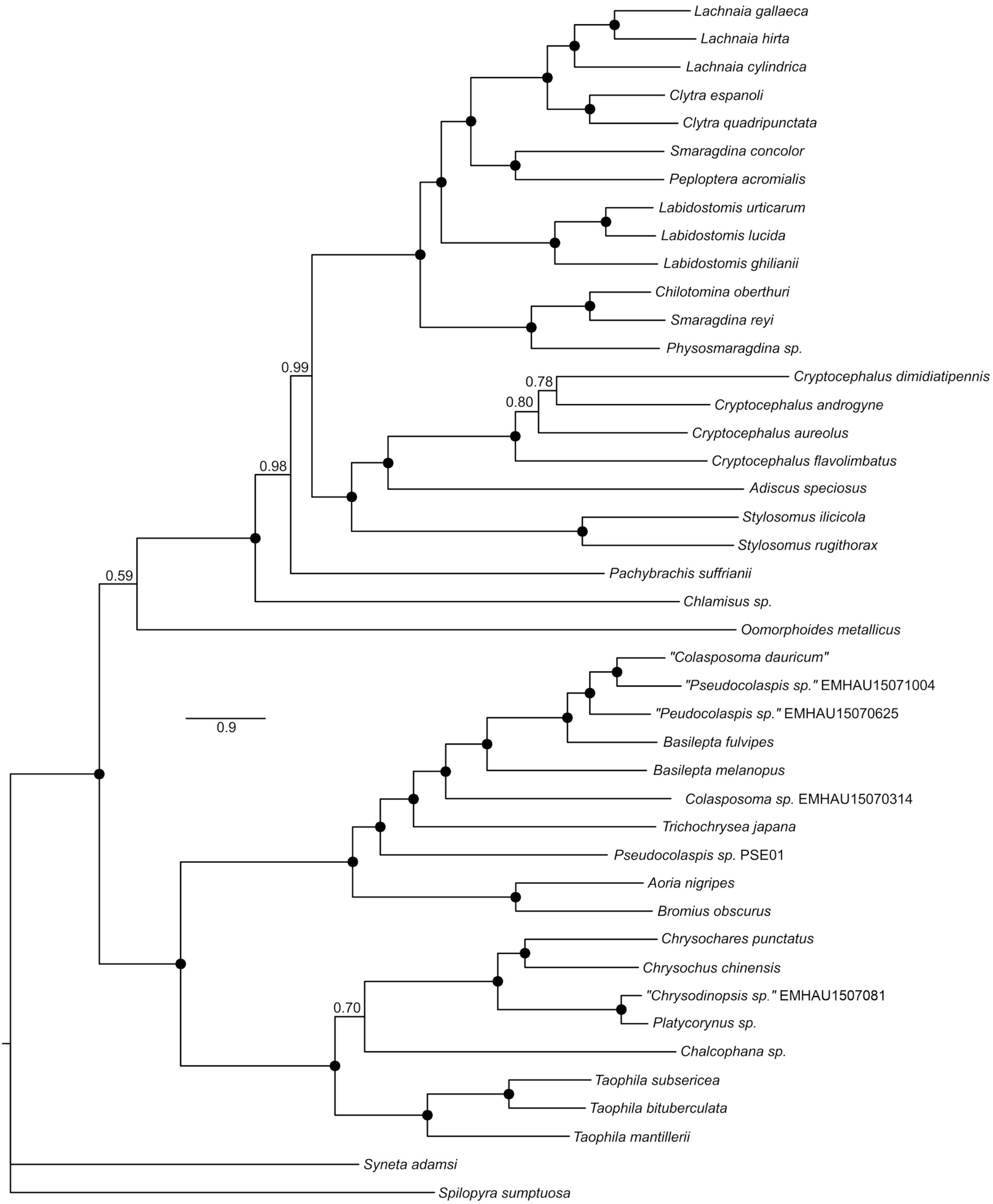

Lachnaia gallaeca
Lachnaia hirta
Lachnaia cylindrica
Clytra espanoli
Clytra quadripunctata
Smaragdina concolor
Peploptera acromialis
Labidostomis urticarum
Labidostomis lucida
Labidostomis ghilianii
Chilotomina oberthuri
Smaragdina reyi
Physosmaragdina sp.
0.99
0.78
Cryptocephalus dimidiatipennis
Cryptocephalus androgyne
0.80
Cryptocephalus aureolus
Cryptocephalus flavolimbatus
0.98
Adiscus speciosus
Stylosomus ilicicola
Stylosomus rugithorax
Pachybrachis suffrianii
0.59
Chlamisus sp.
Oomorphoides metallicus
"Colasposoma dauricum"
"Pseudocolaspis sp." EMHAU15071004
"Peudocolaspis sp." EMHAU15070625
0.9
Basilepta fulvipes
Basilepta melanopus
Colasposoma sp. EMHAU15070314
Trichochrysea japana
Pseudocolaspis sp. PSE01
Aoria nigripes
Bromius obscurus
Chrysochares punctatus
Chrysochus chinensis
"Chrysodinopsis sp." EMHAU1507081
0.70
Platycorynus sp.
Chalcophana sp.
Taophila subsericea
Taophila bituberculata
Taophila mantillerii
Syneta adamsi
Spilopyra sumptuosa
